# Energy aware resource association in RIS assisted VLC-RF communication network

**DOI:** 10.1371/journal.pone.0327467

**Published:** 2025-07-22

**Authors:** Ashu Taneja, Ali Alqahtani, Nayef Alqahtani

**Affiliations:** 1 Chitkara University Institute of Engineering and Technology, Chitkara University, Punjab, India; 2 Department of Networks and Communications Engineering, College of Computer Science and Information Systems, Najran University, Najran, Saudi Arabia; 3 Department of Electrical Engineering, College of Engineering, King Faisal University, Al-Ahsa, Saudi Arabia; Ajman University, UNITED ARAB EMIRATES

## Abstract

Owing to the rapid surge in the number of intelligent devices and data-intensive applications, the demand for reliable communication support has increased. This has put an undue burden on the radio frequency (RF) based communication which suffers from the main challenge of limited spectrum and access congestion. This is overcome with the use of visible light communication (VLC) that offers broader spectrum and high speed data transmission. However, the finite coverage and susceptibility to blockages in VLC can be avoided with optical reconfigurable intelligent surfaces (ORISs), which alter the wireless propagation channel. This paper presents an integrated VLC-RF communication network aided with ORISs and an RF RIS. To support the network communication a resource association algorithm is proposed that associates a set of ORISs to each VLC access point (AP) and node pair. The impact of power allocation on the system performance is also evaluated. It is observed that the presented hybrid system model achieves sum rate gain of 9.43% with equal power allocation. Also, the proposed association scheme improves the total power consumption by 12.74%.

## 1 Introduction

### 1.1 Motivation

With the surge in wireless devices connected to wireless networks, the radio spectrum resources are becoming limited. With the rise of data intensive applications like online gaming, video streaming, high definition (HD) multimedia streaming with services like Netflix and Youtube, large data sets need to be transmitted and processed [1]. The proliferation of smart sensors, Internet-of-things (IoT) devices, smart phones, smart wearables also accumulate massive data to be transmitted in real-time [2]. This growing amount of data traffic frequently exceeds the capacity of the available spectrum leading to access congestion and a decline in service quality [3]. Moreover, there is a significant upswing in the energy consumption owing to this rapid proliferation [4]. In conventional wireless communication, radio frequency (RF) is used to access the wireless networks. However, to support uninterrupted streaming and real-time sensor data exchange in IoT with reduced energy overhead, novel energy efficient communication solutions and spectrum resources are required [5,6]. Visible light communication (VLC) is emerging as a promising solution. It has huge potential to support the high demand of data traffic and offer huge energy savings [7]. VLC utilises the visible light spectrum to transmit the information using optical signals [8]. Since it serves the dual purpose of illumination and communication with the same power, the power consumption is relatively low [9]. The existing lighting infrastructure have LEDs that act as VLC transmitters. The photodiodes (PDs) are used at the receiver end for VLC signals reception. However, due to its reliance on line-of-sight (LoS) propagation links between the transmitter and receiver, it often results in signal blockages and limited coverage [10]. In this case, VLC-RF coexisting systems are a potential candidate for future communication systems [11]. While RF can offer wide coverage and reduce VLC inter-cell interference, VLC uses the spectrum resources in the visible light band to reduce spectrum congestion of RF communications and enhance energy efficiency.

### 1.2 Related work

The literature includes number of research works on VLC-RF systems. However, there are two variations of VLC-RF systems, namely hybrid VLC-RF systems [12] and aggregated VLC-RF systems [13]. The users can be simultaneously served by VLC access point (AP) and RF AP in case of aggregated VLC-RF system. Whereas in case of hybrid VLC-RF systems, the user is either served by VLC AP or RF AP. Due to the presence of heterogeneous APs in such communications systems, they suffer from the challenges of AP assignment, efficient resource allocation and power control [14]. There are number of research papers that evaluate the performance of VLC-RF networks. For example, [15] investigates an AP assignment problem jointly with power allocation in hybrid VLC-RF network for maximum sum rate in a multi-tier heterogeneous network. The authors in [16] consider the power allocation problem in aggregated VLC-RF system. Based on orthogonal frequency division multiple access (OFDMA) the energy efficiency of the system is maximised with optimal subchannel allocation. For the VLC-RF based wireless systems, the important communication metrics are data rates, spectral efficiency and energy efficiency. To achieve the optimal communication metrics, the signal processing techniques like resource allocation, power allocation, AP assignment need to be considered.

Since the VLC system performance is dependent on the LoS link quality, reconfigurable intelligent surfaces (RISs) have been introduced in VLC systems [17]. RISs are intelligent surfaces or metasurfaces that control the reflection of incoming waves enabling smart radio [18]. These are also called intelligent reflecting surfaces (IRSs). By dynamically controlling the phase shifts of number of RIS reflecting elements, directed beamforming is enabled offering LoS communication links. The RIS controller adjusts the phase shifts so that the wireless channel can be altered. In order to increase the signal strength at the desired receiver, there is constructive interference of reflected signals while signals interfere destructively in other directions [19]. These consume less power owing to no requirement of RF transceiver chains and power amplifiers. Apart from passive IRSs, active IRSs have also been introduced that allow selective amplification of reflected signals [20]. Another variation of IRSs is the STAR-RISs which is simultaneous transmission and reflection RISs. These enable selective reflection to the users in the reflection zone as well as selective transmission to the nodes in the transmission zone simultaneously [21]. The use of RISs in VLC-RF systems have been gaining interest. These enhance the communication performance and improve the energy efficiency. In the RF systems, a number of works focus on the assistance of IRSs for improved performance. The authors in [22] consider an IRS aided MIMO system for maximum sum rate. Through the optimization of base station (BS) precoding and RIS phase shifts, channel overhead is reduced using statistical channel state information (CSI). The IRS technology finds usage in diverse applications like mobile edge computing [23], network security [24] and wireless power transfer (WPT) [25]. The assistance of IRSs in the VLC system is elaborated in [26] highlighting the role of RIS technology in addressing the skip-zone problem. The authors in [27] maximize the spectral efficiency in the IRS assisted VLC environment through joint resource utilisation. For focussing the optical power towards a VLC receiver, [28] proposes two types of IRSs based on mirrors and programmable metasurfaces. The orientations of the arrays are adjusted in order to achieve maximum received power gain. The delay in signal transmission from source to the receiver via IRS is calculated in [29] for an IRS-based VLC system. The channel characteristics for the IRS VLC system model are also studied. [30] uses optical IRSs to overcome the challenge of spatial correlation in VLC channels leading to low spatial multiplexing gain. To obtain tradeoff between performance and complexity, the optical IRS are optimised using two algorithms for optimal RIS element alignment and source emission power. The potential of IRSs in free space optical (FSO) systems is highlighted in [31] for high rate secure communication. The comparison of optical IRSs with RF IRSs is also presented.

The performance of hybrid VLC-RF system is investigated in [32] for maximum energy efficiency and sum achievable rate. The allocation of optimal power with optimal association of transceivers optimization yields quality-of-service (QoS) to the multiple users in the system. The system energy efficiency, sum rate and outage performance are evaluated in [33] for aggregated VLC-RF networks through joint power allocation, AP assignment and subchannel allocation. It outperforms the hybrid VLC-RF system and achieves considerable system gains. The performance of aggregated VLC-RF system is elaborated in [13] for maximum energy efficiency subject to transmit power constraints and dimming control. As compared to conventional RF system, the considered system achieves improved data rate. Very few papers have introduced IRSs into the hybrid VLC-RF systems.

In this paper, an integrated VLC-RF system is considered in which multiple ORISs assist the VLC communication while an RF RIS assist the RF communication. The use of RISs dynamically alters the propagation of signals enabling smart radio. The main contributions of the paper are summarised as

- An integrated VLC-RF system is considered to avail the advantages of both VLC and RF communication. VLC offers broad spectrum and high speed communication.
- To mitigate the challenge of finite coverage and signal blockages in VLC as well as effects of wireless channels in RF, ORISs and RF RIS are deployed in the system scenario. These dynamically adjust the signal propagation to enhance the system performance.
- For better utilisation of resources, an association algorithm is proposed which allocates a set of ORISs to each VLC AP-node pair for maximum system sum rate.
- The hybrid VLC-RF system is evaluated for system sum rate and energy efficiency. A power consumption of the hybrid system is also obtained.
- In the end, the impact of power allocation on the system performance is carried out. The performance comparison with the conventional VLC system and RF system is also investigated.

## 2 System model

**Fig 1:**
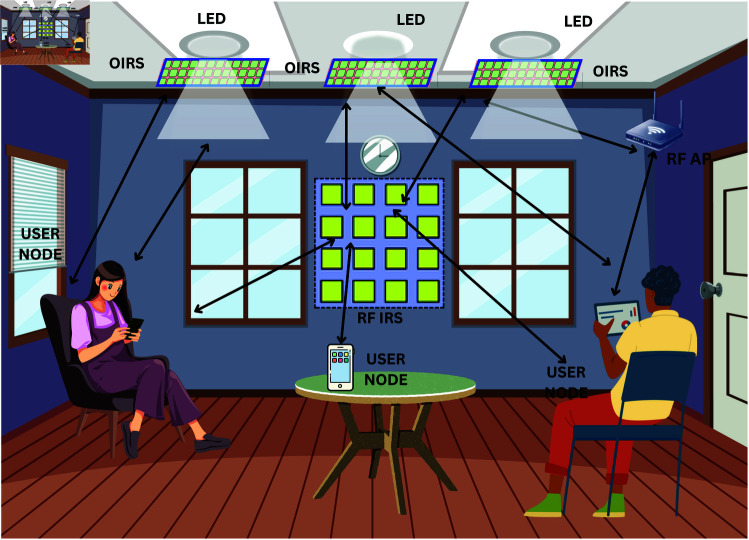
An indoor communication scenario assisted by RF IRS and ORIS RIS.

Consider an indoor communication scenario of VLC-RF system with multiple users as shown in Fig 1. Let the user set is denoted by 𝒦={1,2,....K}. The users are randomly located in the indoor communication system. The indoor lamps act as VLC APs consisting of array of LEDs which are assisted with optical IRSs with *Z* elements arranged as uniform linear array (ULA). There are *V* VLC APs in the indoor system model. The VLC communication is assisted by *L* ORIS units each equipped with *N* reflecting elements. The RF AP is equipped with ULA with *M* antenna elements. The RF communication is assisted by RF IRS with *N* unit ULA elements. In order to enable the synchronization and coordination of RF and VLC APs, they are connected to a centralised control unit. It is assumed that the channel information is known at the central control unit. To enable service from APs simultaneously, each user node has a PD and an antenna.

### 2.1 VLC channel modelling

Suppose the user nodes being served by VLC AP *v* is denoted by a set ${𝒦}_{v}$, with 𝒱={1,2....V} denoting the complete set of VLC APs. To model the VLC communication between node *k* and VLC AP *v*, Lambertian channel model is used as follows

$$h_{k,v}=\left\{ \begin{array}{cc} \frac{(\zeta +1)\mu^{2}A_{k}G}{2\pi r_{k,v}^{2}sin^{2}(\varphi_{0})}cos^{\zeta }(\psi_{k,v})cos(\varphi_{k,v}) & 0\leq \varphi_{k,v}\leq \varphi_{0}\\ 0 & otherwise \end{array}\right.$$ (1)

with *G* denoting the optical filter gain, μ being the refractive index, $\varphi_{0}$ is the semi angle of PD FoV. ζ is the Lambertian index, $r_{k,v}$ is the distance between node *k* and VLC AP *v*, *A*_*k*_ is the PD area on receiver *k*, $\varphi_{k,v}$ is the node *k* PD’s incidence angle with $\psi_{k,v}$ is the source LED array emission angle on VLC AP *v*.

The association between node *k* and VLC AP *v* is defined by an association matrix defined as

$$\lambda_{k,v}=\left\{ \begin{array}{cc} 1 & v=v^{*}\\ 0 & otherwise \end{array}\right.$$ (2)

where ${\text{v}}^{*}$ denotes the closest VLC AP to node k such that $v^{*}=\arg\min_{v}r_{k,v}$.

To represent the ORIS reflected channel path, VLC AP *v* transmission to node *k* is assisted by ${\text{n}}^{\text{th}}$ element of ORIS as follows

$$h_{k,n,v}=\left\{ \begin{array}{cc} \frac{\xi (\zeta +1)\mu^{2}A_{k}G}{2\pi (r_{k,n}+r_{n,v}{)}^{2}sin^{2}(\varphi_{0})}cos^{\zeta }({\bar{\psi }}_{n,v})cos({\bar{\varphi }}_{k,n}) & 0\leq {\bar{\varphi }}_{k,n}\leq \varphi_{0}\\ 0 & otherwise \end{array}\right.$$ (3)

with ξ being the ORIS reflectivity, ${\bar{\psi }}_{k,n}$ is the source emission angle on ORIS unit *k*. The incidence angle on the node *k* from ORIS unit *n* is denoted by ${\bar{\varphi }}_{k,n}$ while *r*_*k*,*n*_ and $r_{n,v}$ be the distance between node *k* to ORIS element *n* and distance between ORIS element *n* to VLC AP *v* respectively. The overall channel gain in the VLC system is given by

$$H_{1}=h_{k,v}\lambda_{k,v}+\sum_{n=1}^{N}h_{k,n,v}f_{v,n,k}$$ (4)

where $f_{v,n,k}$ is an assignment variable whose value is 1 when ${\text{n}}^{\text{th}}$ ORIS element is assigned to serve node *k*. Its value is zero otherwise.

### 2.2 RF channel modelling

In order to model the direct RF channels between RF AP and the nodes, Rayleigh fading channel is used. Denoting the path loss by $\alpha_{0}$ at reference distance of 1 m, $\alpha_{k}$ for the RF AP to node *k* link with distance *r*_*k*_, the direct channel from RF AP to all the nodes is given by

$$G={\left[g_{1},g_{2}......g_{K}\right]}^{T}$$ (5)

with $g_{k}=\sqrt{\alpha_{0}r_{k}^{-\alpha_{k}}}{\bar{g}}_{k}$ with ${\bar{g}}_{k}\in {\mathbb{C}}^{M\times 1}$ representing the Gaussian distributed elements which are independent and identically distributed with zero mean and unit variance.

Since the channels from RF AP to the IRS and IRS to the nodes are LoS channels, so Rician fading model is used to characterise these channels. The channel from RF AP to the ${\text{n}}^{\text{th}}$ element of RIS is given as follows

$${\tilde{g}}_{n}=\sqrt{\alpha_{0}{\hat{r}}^{-\hat{\alpha }}}\left(\sqrt{\frac{\varsigma }{\varsigma +1}}{\tilde{g}}_{n}^{LoS}+\sqrt{\frac{1}{\varsigma +1}}{\tilde{g}}_{n}^{NLoS}\right)$$ (6)

with $\hat{\alpha }$ and $\hat{r}$ being the path loss and distance between RF AP and RF RIS. On similar lines, the channel from RF RIS to the node *k* is

$${\bar{g}}_{k}=\sqrt{\alpha_{0}{\bar{r}}_{k}^{-\bar{\alpha }}}\left(\sqrt{\frac{\varsigma }{\varsigma +1}}{\bar{g}}_{k}^{LoS}+\sqrt{\frac{1}{\varsigma +1}}{\bar{g}}_{k}^{NLoS}\right)$$ (7)

with $\bar{\alpha_{k}}$ and ${\bar{r}}_{k}$ being the path loss and distance between RF RIS to the ${\text{k}}^{\text{th}}$ node. ${\bar{g}}_{k}^{NLoS}\in {\mathbb{C}}^{M\times 1}$ and ${\tilde{g}}_{n}^{NLoS}\in {\mathbb{C}}^{N\times 1}$ follow Gaussian distribution. The channel characteristics from RF AP to RF RIS and that of RF RIS to all the nodes is represented by

$$\bar{G}={\left[{\bar{g}}_{1},{\bar{g}}_{2}......{\bar{g}}_{N}\right]}^{T}$$ (8)

$$\tilde{G}={\left[{\tilde{g}}_{1},{\tilde{g}}_{2}......{\tilde{g}}_{K}\right]}^{T}$$ (9)

### 2.3 RF RIS properties

The properties of RF RIS is represented by a reflection matrix $\Theta =diag(\theta_{1},\theta_{2},.....\theta_{N})$ where $\theta_{1},\theta_{2},.....\theta_{N}$ represent the reflection coefficients of the *N* elements of RF RIS. Each element reflection coefficient is denoted by $\theta_{n}=A_{n}e^{j\phi_{n}}$ with amplitude coefficient *A*_*n*_ and phase shift $\phi_{n}$. Thus, $\Theta =diag(A_{1}e^{j\phi_{1}},A_{2}e^{j\phi_{2}}......A_{N}e^{j\phi_{N}})$ and $\phi_{n}\in [-\pi ,\pi ]$ and $A_{n}\in [0,1]$. The total channel gain is the sum of direct channel and RF RIS reflected channel given as

$$H_{2}=\tilde{G}\Theta \bar{G}+G$$ (10)

### 2.4 System achievable rate

The system achievable rate is the sum of achievable rate of VLC communication system and RF communication system given as

$$R_{T}=\sum_{k=1}^{K}R_{VLC}+R_{RF}$$ (11)

where $R_{VLC}$

$$R_{VLC}=\sum_{k=1}^{K}R_{v,k}$$ (12)

$$R_{v,k}=\frac{B_{1}}{2}log_{2}(1+\frac{e}{2\pi }\gamma_{v,k})$$ (13)

with $\gamma_{k,v}$ being the SINR given by

$$\gamma_{v,k}=\frac{H_{1}^{2}p_{v}}{\sum_{v^{\prime }=1}^{V}h_{k,v^{\prime }}^{2}(1-\lambda_{k,v^{\prime }})p_{v^{\prime }}+\eta_{1}B_{1}}$$ (14)

where $p_{v}$ is the power allocated to VLC AP *v*, *B*_1_ is the VLC communication bandwidth and $\eta_{1}$ is the noise power spectral density (PSD).

$$R_{RF}=B_{2}log_{2}\left(1+\gamma_{RF,k}\right)$$ (15)

where $\gamma_{RF,k}=\frac{\rho_{k}}{\eta_{2}B_{2}}$, $\rho_{k}$ is the power received at ${\text{k}}^{\text{th}}$ node such that the power vector is represented as $\rho =[\rho_{1},\rho_{2}.....\rho_{K}{]}^{T}$, $\eta_{2}$ is the noise PSD and *B*_2_ is the bandwidth of RF communication.

To carry out the energy efficiency of the system, it is required to obtain the total power consumption as defined below.

$$\epsilon =\frac{R_{T}}{P_{tot}}$$ (16)

The total power consumption *P*_*tot*_ is the sum of hardware power consumption *P*_*H*_ and transmit power consumption *P*_*Tx*_.

$$P_{tot}=P_{Tx}+P_{H}$$ (17)

$$P_{H}=VP_{1}+P_{2}+NP_{3}+LNP_{4}$$ (18)

where *P*_1_ is the power consumed by VLC AP, *P*_2_ is static power consumption of RF AP, *P*_3_ denotes the power consumed by each element of RF RIS while *P*_4_ represents the power consumption of ORIS element.

The transmit power consumption *P*_*Tx*_ is given by

$$P_{Tx}=P_{Tx,1}+P_{Tx,2}$$ (19)

$$P_{Tx,1}=tr(Q\rho Q^{H})$$ (20)

where *Q* is the zero forcing precoding matrix at RF AP.

$$P_{Tx,2}=\sum_{v=1}^{V}p_{v}$$ (21)

## 3 Association of ORIS with user nodes

In the hybrid VLC-RF communication scenario defined in Sect 2, multiple ORISs are deployed to assist the VLC communication between VLC APs and user nodes. While RF communication between RF AP and the communicating nodes is assisted by RF RIS. This section associates ORIS to each VLC AP-node pair *v*–*k* such that the total achievable rate at the user node due to both VLC and RF communication is maximised. As defined in Sect 2, 𝒦={1,2,....K} denotes the complete user set while 𝒱={1,2....V} denotes the complete set of VLC APs. The the user nodes being served by VLC AP *v* is denoted by a set ${𝒦}_{v}$ and the length of set ${𝒦}_{v}$ is upper bound by a threshold ν. For a particular VLC AP *v* to user node *k* transmission, it selects optimal ORIS ${\text{l}}^{*}$ that offers maximum sum rate at node *k*. The association of optimal ORIS to assist each *v*–*k* communication against all ORISs results in improved system performance. Moreover, the optimal utilisation of resources enhances the network operational efficiency with reduced energy overhead. The algorithm and its steps are given in Algorithm 1.

### 3.1 Proposed association algorithm

Let us suppose ${𝒦}_{1},{𝒦}_{2},.....{𝒦}_{L}$ denotes the user groups assigned to the ORISs {1,2,....L}. Initially each user node *k* in the set {𝒦} selects its nearest VLC AP *v*. This nearest AP will serve as the master AP for node *k* as it offers strong channels for *v*–*k* transmission. It is to ensure that maximum number of user nodes served by each VLC AP and each ORIS should not exceed the defined threshold ν, that is, length $\left\{{𝒦}_{v}\right\}\leq \nu$ and length $\left\{{𝒦}_{l}\right\}\leq \nu$. After that, for each *v*–*k* pair transmission, the SINR $\gamma_{v,k}$ at node *k* is calculated. The RF SINR $\gamma_{RF,k}$ owing to RF communication at node *k* is also obtained. The optimal ORIS ${\text{l}}^{*}$ that maximises the total SINR at node *k* is selected and assigned for particular *v*–*k* pair. The user set ${𝒦}_{l^{*}}$ is updated with the node *k*. This is repeated for all VLC AP and node pairs.

**Fig 2:**
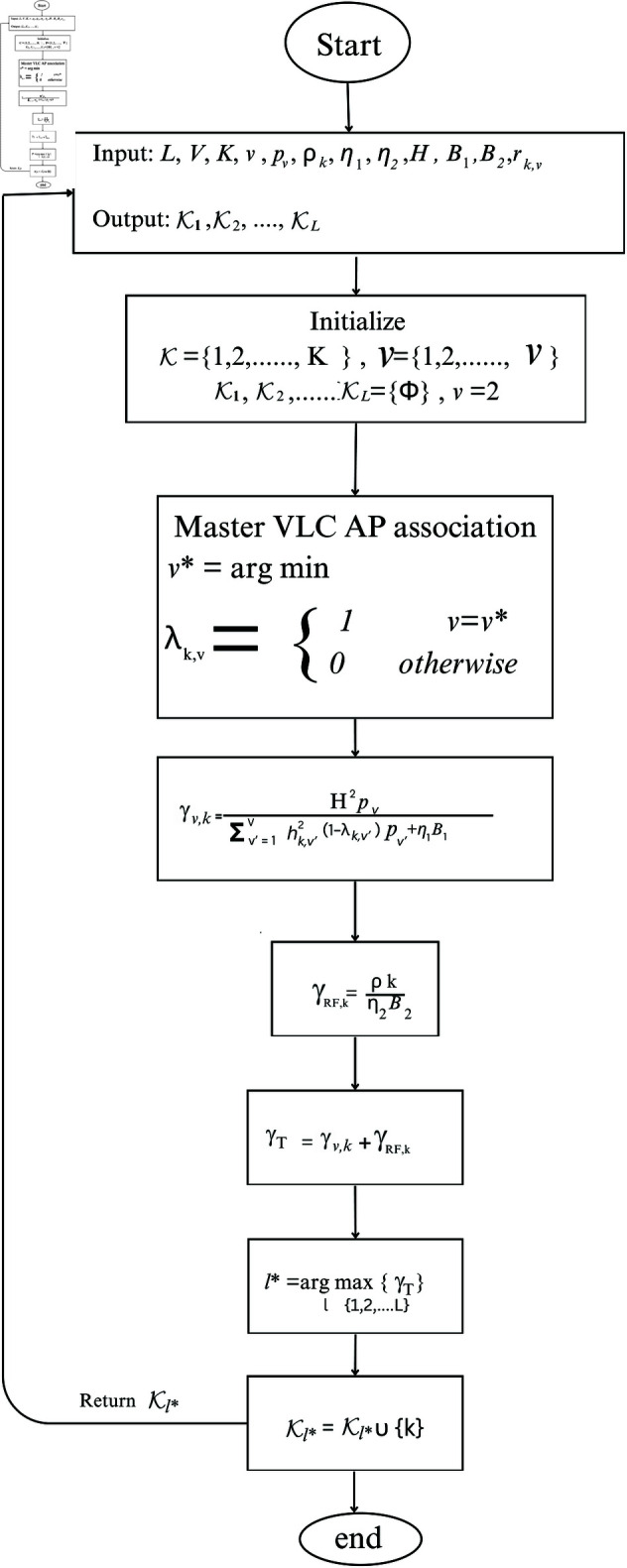
Illustration of the flow of operations in the proposed algorithm.

The Algorithm 1 explains the proposed association approach. The flow of operations associated with the proposed algorithm is summarised in the flowchart shown in Fig 2.

**Algorithm 1. Optimal ORIS association algorithm.**

**Input:** L, V, K, ν, $p_{v}$, $\rho_{k}$, $\eta_{1}$, $\eta_{2}$, H, *B*_1_, *B*_2_, $r_{k,v}$

**Output:** ${𝒦}_{1},{𝒦}_{2},....,{𝒦}_{L}$

1. Initialization

𝒦={1,2,...K}, 𝒱={1,2,...V}, ${𝒦}_{1},{𝒦}_{2},....,{𝒦}_{L}=\left\{\Phi \right\}$, ν=2

2. Master VLC AP association

$v^{*}=\arg\min_{v}\left\{r_{k,v}\right\}$

$\lambda_{k,v}=\left\{ \begin{array}{cc} 1 & v=v^{*}\\ 0 & otherwise \end{array}\right.$

3. Find $\gamma_{v,k}$, for VLC communication for each (v–k) pair

$\gamma_{v,k}=\frac{H^{2}p_{v}}{\sum_{v^{\prime }=1}^{V}h_{k,v^{\prime }}^{2}(1-\lambda_{k,v^{\prime }})p_{v^{\prime }}+\eta_{1}B_{1}}$

4. Find $\gamma_{RF,k}$ for RF communication

$\gamma_{RF,k}=\frac{\rho_{k}}{\eta_{2}B_{2}}$

5. Find total SINR at each node

$\gamma_{T}=\gamma_{v,k}+\gamma_{RF,k}$

6. For each (v–k) pair, select optimal ORIS l^*^

$l^{*}=\arg\max_{l\in \left\{1,2,....L\right\}}\left\{\gamma_{T}\right\}$

7. Associate that node to the user set served by optimal ORIS

l^*^

${𝒦}_{l^{*}}={𝒦}_{l^{*}}\cup \left\{k\right\}$

8. Repeat for all the v–k pairs

**return** ${𝒦}_{l^{*}}$

The flowchart starts with defining the input and output parameters. The first step is the initialization of the parameters and threshold value. The next step is the master VLC AP association in which each user node *k* finds its master VLC AP ${\text{v}}^{*}$. After that, the SINR at each node is obtained owing to both VLC communication $\gamma_{v,k}$ and RF communication $\gamma_{RF,k}$. The total SINR at each node is calculated as $\gamma_{T}$.The ORIS ${\text{l}}^{*}$ that maximizes $\gamma_{T}$ is obtained. In the end, that node is assigned to the subset ${𝒦}_{l^{*}}$. The output is returned.

## 4 Results and discussions

This section presents the numerical results of the hybrid VLC-RF system defined in Sect 2. The hybrid VLC-RF system is simulated in MATLAB to validate the numerical analysis presented in Sect 2. It is considered that each simulation setup run for over 10^4^ iterations which are averaged to obtain the results. The hybrid VLC-RF system is evaluated for performance under proposed resource association scheme. The indoor communication network space has dimensions of 25 m x 25 m x 10 m. There are 4 VLC APs mounted along the indoor corridor at the coordinates (5, 1, 6.5)m, (10, 24, 6.5)m, (15, 1, 6.5)m and (20, 24, 6.5)m. The ORISs are mounted on the four walls (0,12.5, 5)m (12.5,0,5)m, (12.5,0,5)m and (25,12.5,5)m. The RF AP is located at (12.5, 12.5, 8)m with RF RIS at (25,25,8)m. The user nodes are randomly distributed in the indoor space with height 0.85 m above ground plane. Each node has an antenna and a PD for efficient VLC and RF reception. The parameters considered for MATLAB simulations are tabulated in Table 1.

**Table 1:** Different parameters considered for simulation.

Parameters	Value	Parameters	Value
*L*	5	*K*	10
*N*	50	*Z*	20
*G*	1	μ	1.5
$\varphi_{0}$	$70^{\circ }$	ζ	1
*A*_*k*_	1 ${\text{cm}}^{2}$	ξ	0.9
$\alpha_{0}$	–40 dB	$\hat{\alpha }$	2.1
$\alpha_{k}$	4.1	$\bar{\alpha }$	2.2
ς	3dB	*B*_1_	0.5 MHz
*B*_2_	5 MHz	$\eta_{1}$	$10^{-21}\;{\text{A}}^{2}$/Hz
$\eta_{2}$	–174 dBm/Hz	*f*_*c*_	6 GHz
*P*_1_	1 W	*P*_2_	2 W
*P*_3_	0.01 W	*P*_4_	0.01 W

**Fig 3:**
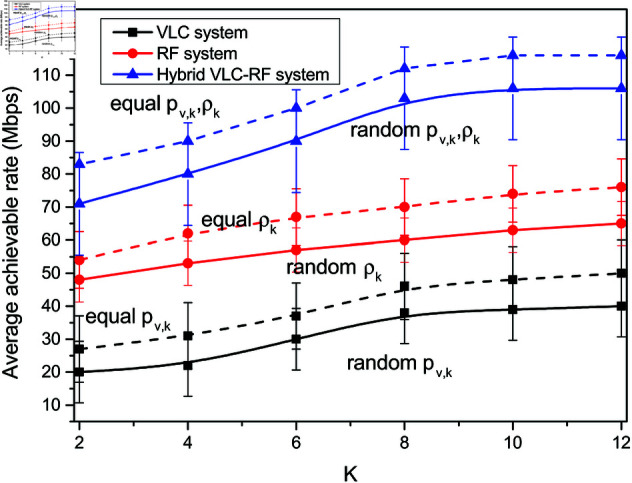
Variation of system achievable rate for different number of users with different power allocation schemes.

The trend of average achievable rate with different number of users in the system is depicted in Fig 3. It considers the variation of three communication system scenarios depicting RF communication, VLC communication and hybrid VLC-RF communication. The hybrid system model supports both RF and VLC communication. The maximum achievable rate is observed in the hybrid system. This is attributed to the availability of both RF AP and VLC AP to which the user nodes connect. It is observed that the achievable rate increases with varying number of users *K*. Increasing the number of user nodes for a given power budget improves the system achievable rate. The VLC communication achieves the least data rate of 20 Mbps with *K* = 2. The impact of power allocation on the rate performance of the three systems is also elaborated. It is shown that equal power allocation scheme outperforms the random power allocation in all the three models. There is an improvement of 9.43% in the rate achieved in the hybrid system with equal power allocation over the random counterpart with *K* = 12. The 95% confidence intervals are also presented for comparison of three system scenarios in terms of average achievable rate.

**Fig 4:**
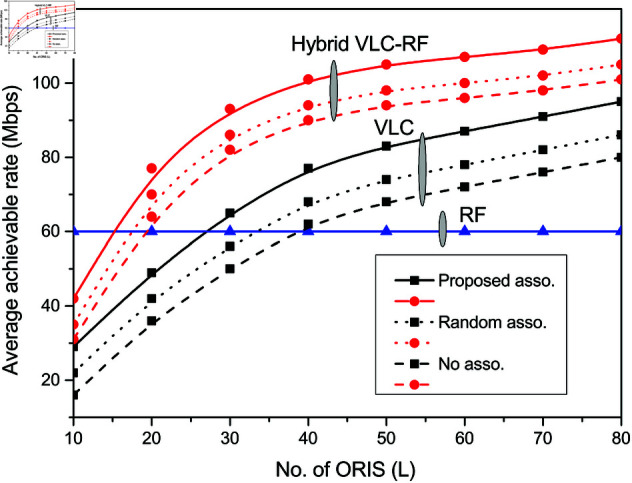
System achievable rate vs L for different association schemes.

Fig 4 shows the variation of system achievable rate with number of ORIS elements *L* with different resource association schemes. It is shown to increase with increasing *L* in both VLC system and hybrid VLC-RF system. The response in the RF system is independent of ORIS elements *L* which is visible by a constant line in blue. The performance of the proposed association algorithm is evaluated and compared with random association scheme and system model incorporating no association. It is observed that the average system rate improves by 10.4% in the VLC system and by 6.66% in the hybrid system with *L* = 80 incorporating proposed association scheme in comparison to random association.

**Fig 5:**
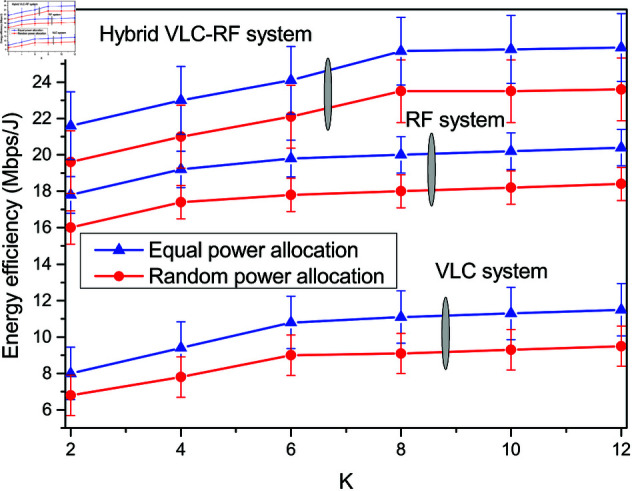
Energy efficiency for different system scenarios for varied K.

The energy efficiency analysis carried out in Sect 2 is validated through simulations in Fig 5. The variation of energy efficiency with the number of varied user nodes *K* is plotted in Fig 5. The system energy efficiency increases with *K* and the hybrid VLC-RF system outperforms the other two system approaches achieving maximum of 23.6 Mbps/J. Moreover, the use of equal power allocation in the hybrid system offers an energy efficiency gain of 9.74%. The same trend is visible in RF communication system and VLC system with variation in *K* and power allocation strategy. It is observed that RF system achieves maximum energy efficiency of 20.4 Mbps/J and VLC system 11.5 Mbps/J with equal power allocation. The plot also gives the confidence intervals to evaluate the energy efficiency comparison system wise.

**Fig 6:**
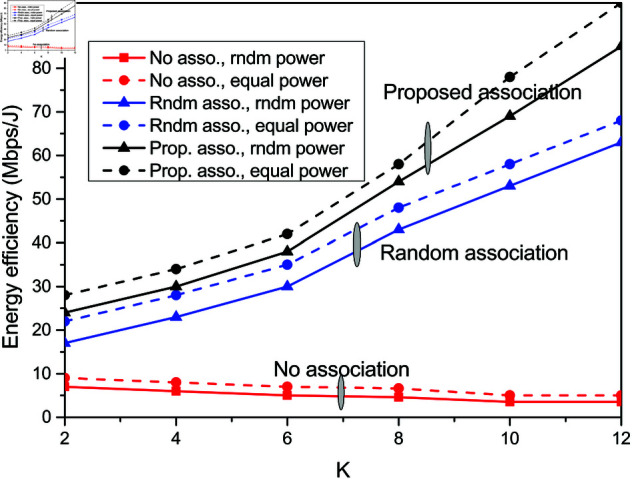
Variation of energy efficiency with K for different associations.

The impact of association algorithm in which ORISs are assigned to different user groups is highlighted in Fig 6. The energy efficiency of the system is shown to improve with the proposed resource association scheme. The association of set of ORISs to support particular AP-node transmission against all the ORISs results in reduced energy consumption and thus increases the energy efficiency. This is evident from Fig 6 that proposed association improves the energy efficiency with varied *K*. Moreover, the impact of power allocation reveals 11.76% gain with equal allocation of power among the user nodes over random user power allocation. In comparison to random ORIS association and no ORIS association, this proposed approach suggests the potential of resource optimisation and power optimisation in improving energy efficiency. In system model not incorporating any resource association, the energy efficiency degrades with more users. This is attributed to increased resource and energy overhead with large user set.

**Fig 7:**
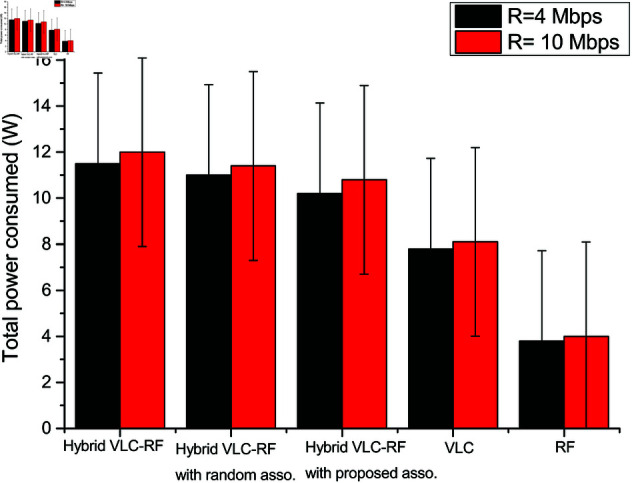
Total power consumption of different system scenarios under different target rates.

Fig 7 shows the total power consumed in the system model utilising VLC communication, RF communication and hybrid VLC-RF communication with and without association schemes. In order to achieve a particular data rate, the power consumption is plotted in Fig 7. It is observed that the hybrid system without incorporating any resource association consumes maximum power. This is due to the obvious reason of increased energy utilisation owing to more resources and associated circuitry. It is observed that to attain 4 Mbps data rate in RF system, the total power consumption is 3.8W while this value stands at 7.8W in VLC system model. Due to the presence of both RF AP and VLC AP in the hybrid system supporting user communication, the total power consumed is 11.5W for achieving 4 Mbps rate. The proposed association scheme improves the total power consumption by 12.74%. The figure also presents the confidence intervals.

## 5 Conclusion

To meet the data traffic demands of proliferating intelligent devices and the significant upswing in the energy consumption owing to this proliferation, an integrated VLC-RF system is presented in this paper. To cope up with the challenges of signal degradation owing to RF and VLC wireless propagation channels, ORISs and RF RIS are utilised for providing extended network coverage. The presented hybrid system is evaluated for achievable sum rate and energy efficiency. A power consumption model is presented in order to carry out the energy efficiency analysis. The impact of power allocation on the system performance is also investigated. It is observed that increasing the user nodes for a given power budget improves the system achievable rate. There is an improvement of 9.43% in the rate achieved in the hybrid system with equal power allocation over the random counterpart with *K* = 12. Further, the optimal association of ORISs to each VLC AP-node pair is presented such that the system sum rate is maximised. For performance comparison, hybrid VLC-RF system with proposed association approach is compared with conventional VLC system and RF systems with no association. The proposed resource control algorithm improves the system rate by 6.66% with *L* = 80. The variation of energy efficiency reveals that it increases with *K* and the hybrid VLC-RF system outperforms the other two system approaches achieving maximum of 23.6 Mbps. In the end, it is shown that the total system power consumption improves by 12.74% with the proposed association algorithm.

## References

[1] Jha S, Ahmad S, Abdeljaber HAM, Othman NA, Nazeer J, Yadav K, et al. Enabling resilient wireless networks: OFDMA-based algorithm for enhanced survivability and privacy in 6G IoT environments. IEEE Trans Consumer Electron. 2024;70(1):3810–9. doi: 10.1109/tce.2024.3370414

[2] Iqal ZM, Selamat A, Krejcar O. A comprehensive systematic review of access control in IoT: requirements, technologies, and evaluation metrics. IEEE Access. 2023.

[3] Jiang W, Han B, Habibi MA, Schotten HD. The road towards 6G: a comprehensive survey. IEEE Open J Commun Soc. 2021;2:334–66. doi: 10.1109/ojcoms.2021.3057679

[4] Saad W, Bennis M, Chen M. Avision of 6G wireless systems: applications, trends, technologies, and open research problems. IEEE Network. 2020;34(3):134–42. doi: 10.1109/mnet.001.1900287

[5] Saluja D, Singh R, Saluja N, Kumar S. Connectivity improvement of hybrid millimeter wave and microwave vehicular networks. IEEE Trans Intell Transport Syst. 2023;24(2):1456–64. doi: 10.1109/tits.2022.3221337

[6] Chopra G. A review on rate splitting multiple access: challenges and future directions. In: 2023 International Conference on Emerging Smart Computing and Informatics (ESCI); 2023. p. 1–7.

[7] Kumari M, Arya V. Integrated long reach and energy-efficient NGPON-VLC system with OCDM/WDM for smart green buildings. Optik. 2023;289:171280. doi: 10.1016/j.ijleo.2023.171280

[8] Matheus LEM, Vieira AB, Vieira LFM, Vieira MAM, Gnawali O. Visible light communication: concepts, applications and challenges. IEEE Commun Surv Tutorials. 2019;21(4):3204–37. doi: 10.1109/comst.2019.2913348

[9] Kumari M, Arya V. Realization of high-speed OWC links by incorporating hybrid OCDMA and MDM scheme for HAP-to-satellite applications. Opt Quant Electron. 2024;56(4). doi: 10.1007/s11082-023-06226-1

[10] Wu X, Safari M, Haas H. Access point selection for hybrid Li-Fi and Wi-Fi networks. IEEE Trans Commun. 2017;65(12):5375–85. doi: 10.1109/tcomm.2017.2740211

[11] Wang F, Yang F, Song J, Han Z. Access frameworks and application scenarios for hybrid VLC and RF systems: state of the art, challenges, and trends. IEEE Commun Mag. 2022;60(3):55–61. doi: 10.1109/mcom.001.2100748

[12] Kong J, Ismail M, Serpedin E, Qaraqe KA. Energy efficient optimization of base station intensities for hybrid RF/VLC networks. IEEE Trans Wireless Commun. 2019;18(8):4171–83. doi: 10.1109/twc.2019.2922611

[13] Ma S, Zhang F, Li H, Zhou F, Alouini M-S, Li S. Aggregated VLC-RF systems: achievable rates, optimal power allocation, and energy efficiency. IEEE Trans Wireless Commun. 2020;19(11):7265–78. doi: 10.1109/twc.2020.3010294

[14] Obeed M, Salhab AM, Alouini M-S, Zummo SA. On optimizing VLC networks for downlink multi-user transmission: a survey. IEEE Commun Surv Tutorials. 2019;21(3):2947–76. doi: 10.1109/comst.2019.2906225

[15] Aboagye S, Ngatched TMN, Dobre OA, Ibrahim A. Joint access point assignment and power allocation in multi-tier hybrid RF/VLC HetNets. IEEE Trans Wireless Commun. 2021;20(10):6329–42. doi: 10.1109/twc.2021.3073424

[16] Zhang H, Liu N, Long K, Cheng J, Leung VCM, Hanzo L. Energy efficient subchannel and power allocation for software-defined heterogeneous VLC and RF networks. IEEE J Select Areas Commun. 2018;36(3):658–70. doi: 10.1109/jsac.2018.2815478

[17] Eldeeb HB, Naser S, Bariah L, Muhaidat S. Energy and spectral efficiency analysis for RIS-aided V2V-visible light communication. IEEE Commun Lett. 2023;27(9):2373–7. doi: 10.1109/lcomm.2023.3290025

[18] Cheng J, Wang G, Zhang Q. On the optimal discrete phase values of reconfigurable intelligent surfaces. Antennas Wirel Propag Lett. 2024;23(7):2111–4. doi: 10.1109/lawp.2024.3382210

[19] Liu Y, Gao F, Zhang L. Quantization beam analysis and codebook design for one-bit reconfigurable intelligent surface. IEEE Wireless Commun Lett. 2024;13(7):1793–7. doi: 10.1109/lwc.2024.3388273

[20] Yang X, Wang H, Feng Y. Sum rate maximization for active RIS-aided uplink multi-antenna NOMA systems. IEEE Wireless Commun Lett. 2023;12(7):1149–53. doi: 10.1109/lwc.2023.3264516

[21] Zhao B, Zhang C, Yi W, Liu Y. Ergodic rate analysis of STAR-RIS aided NOMA systems. IEEE Commun Lett. 2022;26(10):2297–301. doi: 10.1109/lcomm.2022.3194363

[22] Zhang H, Ma S, Shi Z, Zhao X, Yang G. Sum-rate maximization of RIS-aided multi-user MIMO systems with statistical CSI. IEEE Trans Wireless Commun. 2023;22(7):4788–801. doi: 10.1109/twc.2022.3228910

[23] Li N, Hao W, Zhou F, Chu Z, Yang S, Muta O, et al. Min–max latency optimization for IRS-aided cell-free mobile edge computing systems. IEEE Internet Things J. 2024;11(5):8757–70. doi: 10.1109/jiot.2023.3322751

[24] Wang Y, Shi W, Huang M, Shu F, Wang J. Intelligent reflecting surface aided secure transmission with colluding eavesdroppers. IEEE Trans Veh Technol. 2022;71(9):10155–60. doi: 10.1109/tvt.2022.3179392

[25] Shi W, Wu Q, Xiao F, Shu F, Wang J. Secrecy throughput maximization for IRS-aided MIMO wireless powered communication networks. IEEE Trans Commun. 2022;70(11):7520–35. doi: 10.1109/tcomm.2022.3212734

[26] Aboagye S, Ndjiongue AR, Ngatched TMN, Dobre OA, Poor HV. RIS-assisted visible light communication systems: a tutorial. IEEE Commun Surv Tutorials. 2023;25(1):251–88. doi: 10.1109/comst.2022.3225859

[27] Sun S, Yang F, Song J, Han Z. Joint resource management for intelligent reflecting surface–aided visible light communications. IEEE Trans Wireless Commun. 2022;21(8):6508–22. doi: 10.1109/twc.2022.3150021

[28] Abdelhady AM, Salem AKS, Amin O, Shihada B, Alouini M-S. Visible light communications via intelligent reflecting surfaces: metasurfaces vs mirror arrays. IEEE Open J Commun Soc. 2021;2:1–20. doi: 10.1109/ojcoms.2020.3041930

[29] Abdelhady AM, Amin O, Salem AKS, Alouini M-S, Shihada B. Channel characterization of IRS-based visible light communication systems. IEEE Trans Commun. 2022;70(3):1913–26. doi: 10.1109/tcomm.2022.3143142

[30] Sun S, Mei W, Yang F, An N, Song J, Zhang R. Optical intelligent reflecting surface assisted MIMO VLC: channel modeling and capacity characterization. IEEE Trans Wireless Commun. 2024;23(3):2125–39. doi: 10.1109/twc.2023.3295793

[31] Jamali V, Ajam H, Najafi M, Schmauss B, Schober R, Poor HV. Intelligent reflecting surface assisted free-space optical communications. IEEE Commun Mag. 2021;59(10):57–63. doi: 10.1109/mcom.001.2100406

[32] Wang F, Yang F, Pan C, Song J, Han Z. Hybrid VLC-RF systems with multi-users for achievable rate and energy efficiency maximization. IEEE Trans Wireless Commun. 2023;22(9):6157–70. doi: 10.1109/twc.2023.3239840

[33] Aboagye S, Ngatched TMN, Dobre OA, Poor HV. Energy-efficient resource allocation for aggregated RF/VLC systems. IEEE Trans Wireless Commun. 2023;22(10):6624–40. doi: 10.1109/twc.2023.3244871

